# Synaptic Plasticity Changes in the Somatosensory Cortex During Amyotrophic Lateral Sclerosis Progression and After Swim Training in SOD1-G93A Mice

**DOI:** 10.1007/s12035-026-05979-6

**Published:** 2026-06-04

**Authors:** Anbarieh Saadat, Małgorzata Jasińska, Bartosz Cedro, Alicja Piekarska, Damian J. Flis, Wiesław Ziółkowski, Elżbieta Pyza

**Affiliations:** 1 Department of Cell Biology and Imaging, Jagiellonian University, Kraków, Poland; 2 Department of Histology, Jagiellonian University Medical College, Kraków, Poland; 3 Department of Pharmaceutical Pathophysiology, Medical University of Gdańsk, Gdańsk, Poland; 4 Department of Rehabilitation Medicine, Medical University of Gdańsk, Gdańsk, Poland

**Keywords:** Amyotrophic lateral sclerosis, Synaptic plasticity, Transmission electron microscopy, Swim training, Excitatory/inhibitory ratio, Somatosensory cortex

## Abstract

Somatosensory cortex hyperexcitability is present in the pre-symptomatic stage of amyotrophic lateral sclerosis (ALS) as evidenced by brain recordings, but its synaptic basis remains unclear. We examined synaptic plasticity, the density of asymmetric (putative excitatory) and symmetric (putative inhibitory) synapses, dendritic spine morphology, and the putative excitatory/inhibitory (E/I) ratio in the B2 barrel of the somatosensory cortex in female mice of an ALS mouse model. Transgenic mice, B6SJL-Tg (SOD1*G93A)1Gur/J, were used as the ALS model, and wild-type (WT) B6SJL/F1 mice served as controls. ALS mice were allocated to experimental groups based on disease stage (pre-symptomatic, onset, or terminal) and training condition (swim-trained or untrained). Swim training was applied after the first onset of symptoms (clinical score 1). We analyzed and quantified the density of asymmetric (putative excitatory) and symmetric (putative inhibitory) synapses and E/I ratios using serial electron micrographs to understand how these parameters change during disease progression and whether swim training influences this process. Our results showed stage-dependent alterations in asymmetric (putative excitatory) and symmetric (putative inhibitory) synaptic architecture in ALS. The obtained data showed an increase in the excitatory synaptic density in the presymptomatic ALS mice. This finding is consistent with previous reports of early cortical hyperexcitability and may reflect structural alterations associated with an initial increase in excitatory synapses before disease onset. Importantly, we report here an increase in inhibitory synapses at disease onset. TEM-based synaptic density quantification revealed reduced excitatory synapse density in the B2 barrel of the somatosensory cortex of trained ALS mice compared to WT controls, alongside a trend toward a reduced putative excitatory/inhibitory synaptic ratio. However, as no significant differences were detected between trained and untrained ALS mice, the contribution of swim training to these alterations remains unclear. Notably, swim training was not associated with detectable adverse effects on somatosensory cortex ultrastructure, excitatory synapse density, or the putative excitatory/inhibitory ratio, supporting previous observations that swim training is well tolerated under these experimental conditions. To our knowledge, these results provide the first TEM-based ultrastructural characterization of synaptic architecture in swim-trained SOD1-G93A mice, although further studies are needed to establish the underlying mechanisms and therapeutic relevance in ALS.

## Introduction

Amyotrophic lateral sclerosis (ALS) or Lou Gehrig’s disease is a fatal and progressive neurodegenerative disorder characterized by the degeneration of upper motor neurons in the cerebral cortex and lower motor neurons in the spinal cord. It leads to muscle atrophy, weakness, paralysis, and ultimately death from respiratory failure, typically within 2–5 years from diagnosis [10, 14]. Clinical and preclinical evidence indicate cortical hyperexcitability as a common early feature of ALS, which is not static but evolves as the disease progresses [46]. This process is characterized by an imbalance between cortical excitation and inhibition, which worsens over time and correlates with clinical measures of disease severity [36, 37]. Cortical hyperexcitability is a crucial early event in ALS that is not restricted to the motor cortex, as the somatosensory cortex also shows signs of hyperexcitability, indicating that ALS is a multisystem disorder [8, 25, 36]. The motor and somatosensory cortex are anatomically and functionally connected and play crucial roles in coordinated movement and sensory processing [42]. Previous studies have reported correlated hyperexcitability in motor and sensory cortices, suggesting functional interplay between these regions rather than a strictly unidirectional effect [25, 48]. In rodents, the layer IV of the somatosensory cortex, the barrels cortex, is composed of clustered thalamocortical afferents that relay sensory information from whiskers located on the rodent snout and are surrounded by dense walls of neurons [53]. The barrel cortex serves as a fundamental model for understanding how sensory maps are formed, organized, and modified by experience. The distinct, whisker-related patterns from the brainstem to the cortex provide a clear and accessible system for investigating the molecular and cellular mechanisms of neural development and plasticity [16, 34].

Studies on ALS mouse models with the human G93A mutation in *superoxide dismutase 1* (*SOD1*) gene, a common ALS model, have shown that synaptic dysfunction occurs at pre-symptomatic stages. These mice exhibit early deficits in synaptic transmission and in long-term potentiation (LTP) in the motor cortex, a form of synaptic plasticity crucial for learning and memory [12].

Research using mouse models of ALS, which closely mimic human disease progression, has suggested that certain types of physical exercise, particularly swimming, may have neuroprotective effects, improve metabolic function, and potentially slow disease progression. While running-based exercises show varied outcomes, swim training has proved to be a more beneficial physical activity for both sexes, although the specific physiological adaptations may differ [18, 50]. Swimming appears to address the metabolic and behavioral changes characteristic of ALS, including hyperlocomotion and disruptions in glucose, lactate, and copper metabolism within skeletal muscle and the spinal cord [3, 10, 14, 15]. It has significant neuroprotective and behavioral effects, which are linked to changes in both the motor and somatosensory systems. It has been shown to delay disease onset and extend lifespan in ALS mice [13, 15]. These benefits appear to be mediated by adaptations in the spinal cord and the cerebral cortex, influencing both motor and sensory pathways [4, 15]. While swimming has demonstrated beneficial effects on motor systems and may modulate dysfunctional neural circuitry in ALS [13, 15], it remains unclear whether it directly affects cortical processing in the somatosensory cortex or to what extent it modulates dysfunctional sensory-motor networks.

Although ALS primarily affects motor neuronal networks, the somatosensory cortex is anatomically and functionally interconnected with the motor cortex and exhibits early hyperexcitability. Given that physical exercise modulates synaptic plasticity, we investigated whether swim training affects cortical synaptic architecture in the somatosensory cortex. Studying neuroplasticity, E/I ratios, and synaptic densities in the barrel cortex of ALS mouse models is important for understanding disease mechanisms beyond motor neuron degeneration. Sensory input from whiskers, processed in the barrel cortex, feeds into motor networks and may drive compensatory plasticity or changes consistent with increased neuronal activity, thereby influencing behavioral outcomes such as swimming performance. This approach could also help identify sensory-driven therapeutic targets, such as the modulation of interneuron populations to restore E/I balance and may clarify whether sensory-motor hubs contribute to disease propagation but are underrepresented in ALS research due to its motor-centric focus.

In this study, a female ALS mouse model with SOD1-G93A mutation was used to investigate the progression of ALS in layer IV of the somatosensory cortex, focusing on synaptic plasticity of asymmetric (putative excitatory) and symmetric (putative inhibitory) synapses and dendritic spine plasticity in the B2 barrel.

## Materials and Methods

### Animals

The animals were housed and trained at the Tri-City University Animal House–Research Service Centre–Medical University of Gdansk. All experimental procedures were reviewed and approved by the Local Ethical Committee for Experiments on Animals in Bydgoszcz (decision number 3/2022, 14 February 2022) and the Polish Ministry of the Environment (decision number 141/2017, 11 September 2017, and decision number 174/2021, 01 December 2021). All experiments were carried out in accordance with the European Union Directive 2010/63/EU on the protection of animals used for scientific purposes, with efforts made to minimize animal suffering.

Transgenic female mice expressing human SOD1 with the G93A substitution, B6SJL-Tg(SOD1*G93A) 1Gur/J (ALS mice) (*n* = 26) and wild-type (WT) female mice B6SJL/F1 (*n* = 20) were acquired from the Jackson Laboratory (Bar Harbor, ME, USA). The WT controls were age-matched to transgenic animals to ensure comparability across disease stages. Due to colony management and animal availability constraints, littermate WT controls were not consistently available for all experimental groups and disease stages. Therefore, age- and background-matched WT mice were used in the present study. All the mice were maintained on a B6SJL/F1 background and housed under identical conditions within the same facility. Given the well-characterized phenotype of the SOD1-G93A model, age-matched WT animals provide an appropriate control which has been used in previous studies [3, 10, 15, 18]. In this study, female mice were used for mechanistic studies and therapy testing because they exhibit slower disease progression than males, thereby allowing a longer observation window before reaching the disease endpoint [9].

Animals had free access to standard mouse chow and water (ad libitum) and were housed under controlled laboratory conditions: temperature of 23 ± 1 °C and in a 12 h of light and 12 h of dark cycle (LD12:12). After a 2-week adaptation period, the mice were randomly divided into the following groups according to disease progression and training status: ALS before (*n* = *5*), mice without any signs of the disease; ALS onset (*n* = *7*), mice showing the first symptoms of the disease; ALS terminal untrained (*n* = *7*), and ALS terminal trained (*n* = *7*). Corresponding age-matched groups of WT mice were also formed: WT before (*n* = *5*), WT onset (*n* = *5*), WT untrained (*n* = *5*), and WT swim trained (*n* = *5*).

The clinical score assessment [23] was used to determine disease progression and the time point for animal sacrifice. The clinical score was evaluated based on an 8-point scale depending on the signs exhibited to determine the severity of the disease: no evidence of disease, score 0; shaking of the hindlimbs when suspended by the tail, score 1; weakness in one hindlimb, score 1.5; change in gait, score 2; severe weakness in one hindlimb, score 2.5; extreme weakness in both hindlimbs, score 3; functional paralysis in one hindlimb, score 3.5; functional paralysis in both hindlimbs but the animal can right itself in less than 20 s after being placed on its side, score 4; and finally, when the animal cannot right itself within 20 s after being placed on its side, score 5 (endpoint, followed by euthanasia) according to Hamadeh et al. [23] and Flis et al. [18]. The mice were sacrificed at the following time points: the pre-symptomatic ALS group (ALS before; 10 weeks of age); ALS onset group, when the first symptoms of the disease were apparent in animals (14 to 16 weeks of age, clinical score—1); ALS terminal group, when the last stage of the disease was apparent in untrained ALS animals (17 to 19 weeks of age, clinical score—5). The mice from the ALS before group, and the corresponding WT group were sacrificed at the same age. The WT onset group, parallel to the ALS onset group, was sacrificed at the same age. The terminal groups: ALS trained, WT trained, and WT untrained groups were euthanized at the same age as the ALS terminal untrained animals, when the sedentary ALS terminal group reached the end stage of the disease (clinical score 5).

### Swim Training Protocol

Starting at the onset of disease symptoms in the ALS swimming group, ALS mice assigned to the swimming group and the corresponding WT mice underwent swim training based on previously published protocols by Deforges et al. [13] and Flis et al. [18]. The swim training sessions took place in a specially designed aquarium divided into twelve cylindrical pools (transparent PVC pipes, 20 cm in diameter and 20 cm in height). Mice were individually placed into the pool for 30 min, five times per week. The training was performed without additional weight, and the water temperature was maintained at 30 °C. The duration of the exercises was adapted to the individual capabilities of the mice in the ALS groups. When animals could not swim for 30 min, they were taken out of the water, and after 1 min, were placed back to continue the exercise. Third flooding ended the exercise for that day. When an animal did not complete  the swimming session for three consecutive days, the training was permanently discontinued.

### Transmission Electron Microscopy

Mice were anesthetized with Morbital (25–30 mg/kg body weight) and perfused through the heart with 20 ml of rinse buffer (0.2% glutaraldehyde and 2% paraformaldehyde in 0.1 M phosphate buffer, pH 7.4) followed by 100–150 ml of fixative (2.5% glutaraldehyde and 2% paraformaldehyde in 0.1 M phosphate buffer, pH 7.4). After perfusion, the brains were removed and immersed in the same fixative for 24 h at 4 °C. After washing in 0.1 M phosphate buffer (pH 7.4), 60 μm tangential vibratome sections were cut from the right barrel cortex. After examining the sections under a stereomicroscope (Nikon Optiphot), the slices containing the barrel field were chosen for further analysis.

The slices were washed in 0.1 M sodium cacodylate buffer (pH 7.4), three times for 5 min each, and postfixed at 4 °C with 1% osmium tetroxide in 0.1 M cacodylate buffer, twice for 1 h, the first time with 1.5% potassium ferrocyanide. They were then washed in distilled water twice for 5 min and stored for 40 min at 4 °C in 70% ethanol containing 1% uranyl acetate. Next, sections were dehydrated in increasing concentrations of ethanol (50, 70, 90, and 96%), 5 min each, and in 100% ethanol three times for 5 min. The slices were then washed twice for 10 min in propylene oxide and incubated in Epon resin and propylene oxide mixtures and finally embedded in Epon resin (Polysciences) between two Aclar films. The slices were then photographed (2 × objective magnification) under a light microscope (Nikon Optiphot) equipped with a digital camera (Nikon DXM 1200 F). The images of the barrel fields were stacked and reconstructed using Adobe Photoshop CS5 (Adobe Systems). To prepare the slices that contained the barrel field (layer IV), we used the standard method for transmission electron microscopy (TEM) previously described in Kirov et al. [31] and Knott et al. [32]. The region of the B2 barrel, as previously described by Jasinska et al. [26], was identified using Adobe Photoshop CS5 (Adobe Systems) and the pattern of blood vessels indicating its location within the barrel field.

A series of 4–12 successive sections (65 nm thick) from each sample were cut, collected on formvar-coated copper single-slot grids, and contrasted with 2% uranyl acetate and 0.3% lead citrate. The central regions of the B2 barrel, layer 4, in which cell bodies are sparse, were chosen, and synapses were observed and imaged at 4 k using a JEOL JEM-2100 HT transmission electron microscope (JEOL, Japan). Ten to eighteen serial electron micrographs were taken from successive sections for a 3D reconstruction of dendritic spines in Blender software (version 4.5.3). All micrographs were aligned and stacked in Adobe Photoshop CS5 software. A detailed description of the sample preparation for TEM imaging and the 3D reconstruction of dendritic spines is provided in Saadat et al. [43].

### Quantitative Analysis of Synapses

The center of the B2 barrel was selected for this experiment and synaptic counting because it has a lower cell body density, which minimizes synaptic occlusion on finer neuronal elements [32, 52]. The stereological serial dissector approach was used to quantify and analyze the density of symmetric and asymmetric synapses and to quantify dendritic spines that fit within the stack volume [17, 22, 49, 51]. Counting was performed by placing a sample rectangle over the sequence of serial sections and counting each structure just once through the series; only structures that were completely within the rectangle or intersected the left and upper sides of the rectangle were included [43]. Electron micrographs from all animals in both ALS and WT groups were used for synaptic density analyses.

In the present study, synaptic density was quantified in the center of the B2 barrel by counting synapses, while dendritic spines fully contained within each image stack were identified and reconstructed. Accurate measurement of spine morphological parameters required complete inclusion of the spine within the image stack. Criteria from Knott et al. [32] were used to identify synapses and dendritic spines. Synapses were identified by the presence of apposed pre- and postsynaptic membranes separated by a distinct synaptic cleft and a presynaptic terminal containing synaptic vesicles. Asymmetric and symmetric synapses were identified and classified according to Jasinska et al. [26, 27] based on synaptic symmetry and vesicle morphology. Asymmetric synapses were characterized by a prominent postsynaptic density and round synaptic vesicles, whereas symmetric synapses displayed a thinner postsynaptic density and flattened vesicles, broadly corresponding to excitatory and inhibitory synaptic types, respectively [7, 11, 20, 40, 41]. Hereafter, the terms “excitatory” and “inhibitory” synapses refer to asymmetric and symmetric synapses, respectively.

**Fig. 1:**
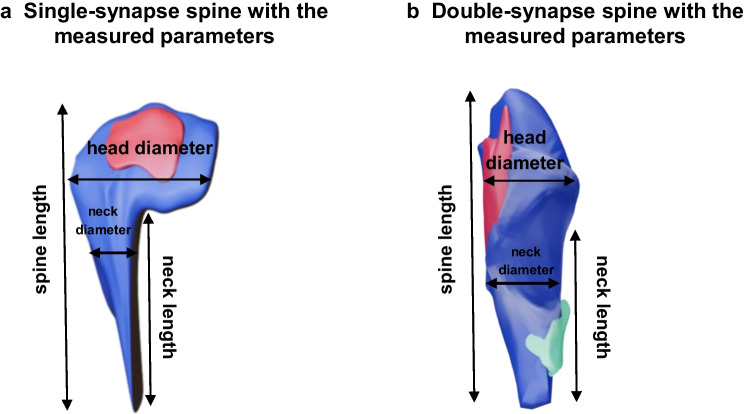
3D reconstruction of single- and double-synapse spines with the measured parameters. **a** Single-synapse spine. The red area indicates an excitatory synapse. **b** Double-synapse spine. The red area indicates an excitatory synapse, and the green area indicates an inhibitory synapse. In both cases, the reconstructed structures correspond to postsynaptic densities (PSDs)

Five animals from each ALS group and three animals from each WT group were randomly selected for dendritic spine quantification. In the electron micrographs, for each spine, the length of the spine, diameter of the spine head and neck (Fig. 1), and the postsynaptic density (PSD) areas of excitatory and inhibitory synapses were measured using Fiji software [47]. The PSD area was determined according to the methodology outlined by Ostroff et al. [39]. The diameter of the spine head was measured at its widest point, aligned with the PSD [6]. Three separate measurements of the neck width at various levels were taken and averaged to establish the neck diameter [2, 28]. Spines were classified as single-synapse spines when they had one excitatory synapse (Fig. 1a), and as double-synapse spines when they had two synapses: one excitatory and one inhibitory (Fig. 1b). The volume of the spine was calculated by adding the area values multiplied by the section thickness across all serial sections where it was present. The morphology of spines was classified according to the criteria described by Harris et al. [24] and modified by Jasinska et al. [28]. Spines were categorized into three distinct shape classifications based on their length (l), diameter of the spine head (dh), and diameter of the neck (dn). Spines that were very long (l ≥ 3 × dn) and exhibited similar diameters for the head and neck (dh ≈ dn) were designated as “thin” spines. Spines characterized by large heads and narrow necks (dh ≥ 2.5 × dn) were referred to as “mushroom” spines. Very short spines, with lengths approximating the diameter of the neck (l ≈ dn), were classified as “stubby” spines. Generally, intermediate spines represented about 30% of single-synapse spines and 16% of double-synapse spines. Due to the significant prevalence of intermediate spines, they were considered distinct spine shapes in addition to stubby, thin, and mushroom spines.

### Statistical Analysis

All data were analyzed using GraphPad Prism 5.0 software (GraphPad Software Inc., USA). To compare the effect of swim training and the progression of disease on synaptic density, we quantified the number of synapses in the volume of more than 100 μm^3^ of the B2 barrel hollow region for each animal and calculated the mean values. The normality of distribution and homogeneity of variances were tested to decide which statistical tests should be used. Data from the excitatory and inhibitory synaptic densities and the differences in the E/I ratio were analyzed using a two-way analysis of variance (ANOVA) to evaluate the effects of genotype (ALS vs WT) and disease progression stage (before, onset, terminal untrained, and terminal trained) within the ALS groups. When significant main or interaction effects were detected, Bonferroni post hoc multiple comparison tests were performed. The PSD area of excitatory and inhibitory synapses, dendritic spine length, neck length, and neck and head diameters were analyzed using one-way ANOVA. When a significant effect was detected, Tukey’s post hoc multiple comparison tests were conducted. To test the relationships between the PSD areas of excitatory and inhibitory synapses and spine head diameters that changed over the course of the disease and after swim training, Pearson’s correlation coefficient was used. The data in the Results text and graphs are presented as mean ± standard error of the mean (SEM) throughout. The results were considered statistically significant with *p* < 0.05.

## Results

**Fig. 2:**
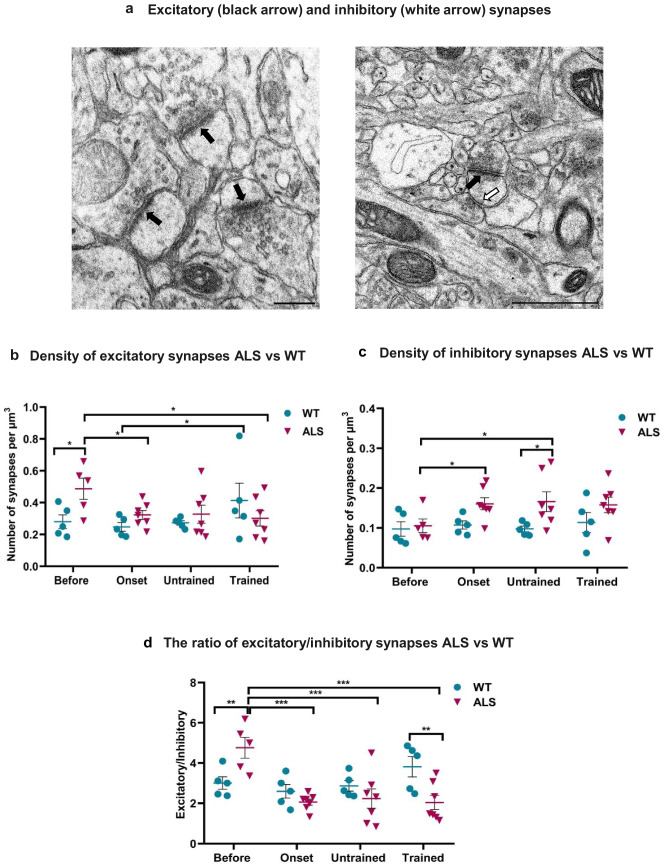
Density of excitatory and inhibitory synapses and the excitatory/inhibitory (E/I) ratio. **a** Electron micrographs taken from the B2 barrel showing single-synapse spines in the left panel and a double-synapse spine in the right panel. Excitatory synapses are indicated by black arrows and inhibitory synapses by white arrows (scale bars: left image, 0.3 μm; right image, 1 μm). **b** Density of excitatory synapses in ALS mice at the pre-symptomatic stage (ALS before, clinical score 0; *n* = 5), disease onset (ALS onset, clinical score 1; *n* = 7), and terminal stage (ALS untrained and ALS trained, clinical score 5; *n* = 7 each), compared with their respective WT groups (WT before, WT onset, WT untrained, and WT trained; *n* = 5 each). Two-way ANOVA: genotype *F*(1, 38) = 2.52, *p* = 0.118; disease stage *F*(3, 38) = 1.75, *p* = 0.17; genotype × stage interaction *F*(3, 38) = 3.44, *p* = 0.024. Bonferroni post hoc tests; *p < 0.05. **c** Density of inhibitory synapses in ALS mice at different disease stages and in the corresponding WT groups (group sizes as in **b**). Two-way ANOVA: genotype *F*(1, 38) = 13.57, *p* = 0.0006; disease stage *F*(3, 38) = 1.89, *p* = 0.144; genotype × stage interaction *F*(3, 38) = 1.18, *p* = 0.325. Bonferroni post hoc tests; *p < 0.05. **d** Ratio of excitatory to inhibitory synapses in ALS and WT groups (group sizes as in **b**). Two-way ANOVA: genotype *F*(1, 38) = 1.47, *p* = 0.231; disease stage *F*(3, 38) = 8.00, *p* = 0.0002; genotype × stage interaction *F*(3, 38) = 9.21, *p* < 0.0001. Bonferroni post hoc tests; **p < 0.01, ***p < 0.001. The scatter plots show individual data points with means ± SEM

We examined synaptic plasticity in the B2 barrel of layer IV of the somatosensory cortex in the female ALS mouse model and compared the density of excitatory (asymmetric) and inhibitory (symmetric) synapses (Fig. 2a) between ALS and WT mice, while also exploring the effects of swim training on synaptic plasticity throughout disease progression. To test how synapses were affected and to understand the balance between excitatory and inhibitory synapses, the number of excitatory and inhibitory synapses and their ratio (E/I) were calculated. Additionally, morphological changes in dendritic spines were analyzed using various geometric parameters.

## Sampling

Synapses were counted within the sampled tissue volumes. For WT mice, the total sampled volume across all WT groups was 922.0 ± 6.71 µm^3^. The mean sampled volume per animal in each WT subgroup was as follows: WT before, 180.5 ± 58.61 µm^3^; WT onset, 186.5 ± 55.22 µm^3^; WT untrained, 185.7 ± 25.5 µm^3^; and WT trained, 185.2 ± 66.17 µm^3^. For ALS mice, the total sampled volume across all ALS groups was 921.1 ± 6.57 µm^3^. The mean sampled volume per animal in each ALS subgroup was as follows: ALS before, 182.9 ± 94.26 µm^3^; ALS onset, 129.5 ± 32.51 µm^3^; ALS terminal untrained, 133.6 ± 34.79 µm^3^; and ALS terminal trained, 132.9 ± 36.97 µm^3^. The sampling volumes were not significantly different across the ALS groups (one-way ANOVA, *F*(3, 22) = 0.23, *p* = 0.84). Detailed data on the numbers of ultrathin sections, excitatory and inhibitory synapses, and E/I ratios per animal in WT and ALS groups are provided in Tables 1 and 2, respectively. The total numbers of ultrathin sections and 3D-reconstructed single- and double-synapse spines for each group are reported in Table 3.

**Table 1:** Numbers of ultrathin sections, excitatory and inhibitory synapses, and E/I ratios per animal in WT groups

WT	Ultrathin sections	Excitatory (asymmetric)	Inhibitory (symmetric)	E/I ratio
Before (*n* = 5)				
#1	7	19	8	2.38
#2	13	27	11	2.45
#3	23	90	22	4.09
#4	17	56	18	3.11
#5	11	30	10	3.00
Onset (*n* = 5)				
#6	7	18	5	3.60
#7	10	26	13	2.00
#8	11	25	12	2.08
#9	22	118	39	3.03
#10	19	52	37	1.41
Untrained (*n* = 5)				
#11	17	46	19	2.43
#12	9	26	11	2.36
#13	18	60	19	3.16
#14	17	71	19	3.74
#15	17	50	19	2.63
Trained (*n* = 5)				
#16	26	74	16	4.63
#17	11	57	23	2.48
#18	12	48	11	4.36
#19	10	34	7	4.86
#20	15	60	22	2.73

**Table 2:** Numbers of ultrathin sections, excitatory and inhibitory synapses, and E/I ratios per animal in ALS groups

ALS	Ultrathin sections	Excitatory (asymmetric)	Inhibitory (symmetric)	E/I ratio
Before (*n* = 5)				
#1	9	47	14	3.36
#2	8	30	6	5.00
#3	9	49	9	5.44
#4	26	160	42	3.81
#5	9	68	11	6.18
Onset (*n* = 7)				
#6	10	64	32	2.00
#7	9	24	18	1.33
#8	16	110	48	2.29
#9	9	31	12	2.58
#10	10	22	10	2.20
#11	10	35	16	2.19
#12	6	18	10	1.80
Untrained (*n* = 7)				
#13	12	39	25	1.56
#14	10	17	20	0.85
#15	8	45	10	4.50
#16	16	65	28	2.32
#17	8	30	12	2.50
#18	12	61	21	2.90
#19	7	13	13	1.00
Trained (*n* = 7)				
#20	23	56	24	2.33
#21	8	15	13	1.15
#22	8	30	21	1.43
#23	8	17	13	1.31
#24	10	31	21	1.48
#25	8	40	13	3.08
#26	10	63	18	3.50

**Table 3:** Total numbers of ultrathin sections and 3D-reconstructed single- and double-synapse spines for each group

	Total number of ultrathin sections	Double-synapse spine	Single-synapse spine
ALS before (*n* = 5)	75	3	22
ALS onset (*n* = 5)	74	6	13
ALS untrained (*n* = 5)	119	5	35
ALS trained (*n* = 5)	108	2	41
WT untrained (*n* = 3)	66	3	22
WT trained (*n* = 3)	70	1	23

## Quantitative Analysis of Synaptic Density

### Total Density of Excitatory Synapses

A two-way ANOVA was performed to assess the effects of genotype (WT vs ALS) and disease stage on the density of excitatory synapses. A significant genotype × stage interaction was observed (two-way ANOVA: *F*(3, 38) = 3.44, *p* = 0.024), indicating that the effect of genotype differed across disease stages. No significant main effects of disease stage (two-way ANOVA: *F*(3, 38) = 1.75, *p* = 0.17) or genotype (two-way ANOVA: *F*(1, 38) = 2.52, *p* = 0.118) were detected. These results indicate that differences between WT and ALS mice vary across the examined stages. Bonferroni post hoc tests showed that excitatory synapse density was significantly higher in the ALS before group than in the corresponding WT group (*p* < 0.05). The density of excitatory synapses was significantly reduced in the ALS onset group in comparison to the ALS before group (*p* < 0.05). There was also a significant decline in excitatory synapse density in the ALS terminal trained mice compared with ALS before (*p* < 0.05). Although a similar downward trend was visible between ALS before and ALS untrained groups, this difference did not reach statistical significance. Within the WT groups, excitatory synapse density was significantly higher in WT trained than in WT onset (*p* < 0.05) (Fig. 2b)*.* This increase suggests training-induced remodelling of cortical synaptic architecture in the WT trained group, reflected by a higher abundance of excitatory synapses.

### Total Density of Inhibitory Synapses

For inhibitory synapse density, two-way ANOVA revealed a significant main effect of genotype (*F*(1, 38) = 13.57, *p* = 0.0006), indicating that the ALS model exhibited a higher density of inhibitory synapses compared with WT mice. There was no significant effect of disease stage (two-way ANOVA: *F*(3, 38) = 1.89, *p* = 0.144) and no genotype × stage interaction (two-way ANOVA: *F*(3, 38) = 1.18, *p* = 0.325), suggesting that the overall difference between genotypes did not significantly vary across stages. Bonferroni post hoc tests revealed that inhibitory synaptic density was significantly higher in the ALS onset group than in the ALS before group (*p* < 0.05). In addition, inhibitory synapse density was significantly higher in ALS terminal untrained mice than in the corresponding WT group (*p* < 0.05) and in the ALS before group (*p* < 0.05) (Fig. 2c).

### The Ratio of Excitatory Versus Inhibitory Synapses

The excitatory/inhibitory (E/I) ratio was calculated as the density of excitatory synapses divided by the density of inhibitory synapses. Two-way ANOVA showed a significant genotype × stage interaction (*F*(3, 38) = 9.21, *p* < 0.0001), demonstrating that genotype-related differences varied depending on disease stage. There was a significant main effect of disease stage (two-way ANOVA: *F*(3, 38) = 8.00, *p* = 0.0002), indicating that the measured outcome changes across disease stages. No overall main effect of genotype was detected (two-way ANOVA: *F*(1, 38) = 1.47, *p* = 0.231). Between the before groups, the E/I ratio in ALS was significantly higher than in the corresponding WT group (*p* < 0.01). In the ALS groups, the E/I ratio at the before stage was significantly higher than at onset and at the terminal stage in both untrained and trained mice (*p* < 0.001 for all comparisons). Within trained groups, the E/I ratio was significantly higher in WT trained than in ALS terminal trained (*p* < 0.01). The E/I ratio was higher in the ALS before group compared with WT, whereas in the trained groups the pattern was reversed, with a lower E/I ratio in ALS than in WT (Fig. 2d).

### Dendritic Spine and Postsynaptic Density Measurments

#### Area of the Postsynaptic Density (PSD)

**Fig. 3:**
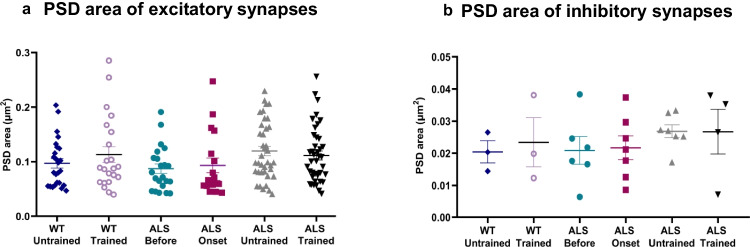
The PSD area of synapses on dendritic spines. **a** PSD area of excitatory synapses on both single- and double-synapse spines. Comparison between WT and ALS groups (ALS before: *n* = 5, ALS onset: *n* = 5, ALS terminal untrained: *n* = 5, ALS terminal trained: *n* = 5, WT untrained and trained groups: *n* = 3 per group); one-way ANOVA: *F*(5, 160) = 1.58, *p* = 0.167. **b** PSD area of inhibitory synapses in ALS mice at the same disease stages and in the corresponding WT untrained and trained groups (group sizes as in panel a); one-way ANOVA: *F*(5, 24) = 0.43, *p* = 0.817. The scatter plots show individual data points with means ± SEM

There was no significant difference in the PSD area of excitatory synapses between ALS groups (one-way ANOVA: *F*(3, 115) = 2.39, *p* = 0.072) and between ALS and WT groups (one-way ANOVA: *F*(5, 160) = 1.58, *p* = 0.167) (Fig. 3a). Similarly, no significant difference was found in the PSD area of inhibitory synapses between ALS groups (one-way ANOVA: *F*(3, 20) = 0.64, *p* = 0.59) and between ALS and WT groups (one-way ANOVA: *F*(5, 24) = 0.43, *p* = 0.817) (Fig. 3b).

#### Total Length of Dendritic Spines and Length of Spine Necks

The length of single- and double-synapse spines did not differ significantly among ALS groups (one-way ANOVA: *F*(3, 110) = 2.24, *p* = 0.087 and *F*(3, 12) = 0.20, *p* = 0.894, respectively). Similarly, no significant differences between ALS and WT groups were observed for the length of single- and double-synapse spines (one-way ANOVA: *F*(5, 153) = 1.62, *p* = 0.157 and *F*(5, 14) = 0.34, *p* = 0.875, respectively).

The length of single- and double-synapse spine necks did not differ significantly among ALS groups (one-way ANOVA: *F*(3, 107) = 0.68, *p* = 0.562 and *F*(3, 12) = 0.42, *p* = 0.737, respectively). Similarly, no significant differences between ALS and WT groups were observed for the neck length of single- and double-synapse spines (one-way ANOVA: *F*(5, 150) = 2.05, *p* = 0.074 and *F*(5, 14) = 0.93, *p* = 0.488, respectively).

#### Diameter of Spine Heads and Necks

Head diameters of single- and double-synapse spines did not differ significantly among ALS groups (one-way ANOVA: *F*(3, 107) = 0.77, *p* = 0.51 and *F*(3, 12) = 0.53, *p* = 0.67, respectively). No significant differences between ALS and WT groups were observed for the head diameter of single- and double-synapse spines (one-way ANOVA: *F*(5, 148) = 0.47, *p* = 0.794 and *F*(5, 15) = 0.35, *p* = 0.871, respectively).

Similarly, neck diameters of single- and double-synapse spines showed no significant differences among ALS groups (one-way ANOVA: *F*(3, 107) = 0.61, *p* = 0.606 and *F*(3, 12) = 0.25, *p* = 0.855, respectively). No significant differences were observed between ALS and WT groups for the neck diameter of single- and double-synapse spines (one-way ANOVA: *F*(5, 148) = 0.75, *p* = 0.584 and *F*(5, 14) = 0.67, *p* = 0.652, respectively).

#### Correlation of the PSD Area of Synapses with Spine Head Diameter

**Fig. 4:**
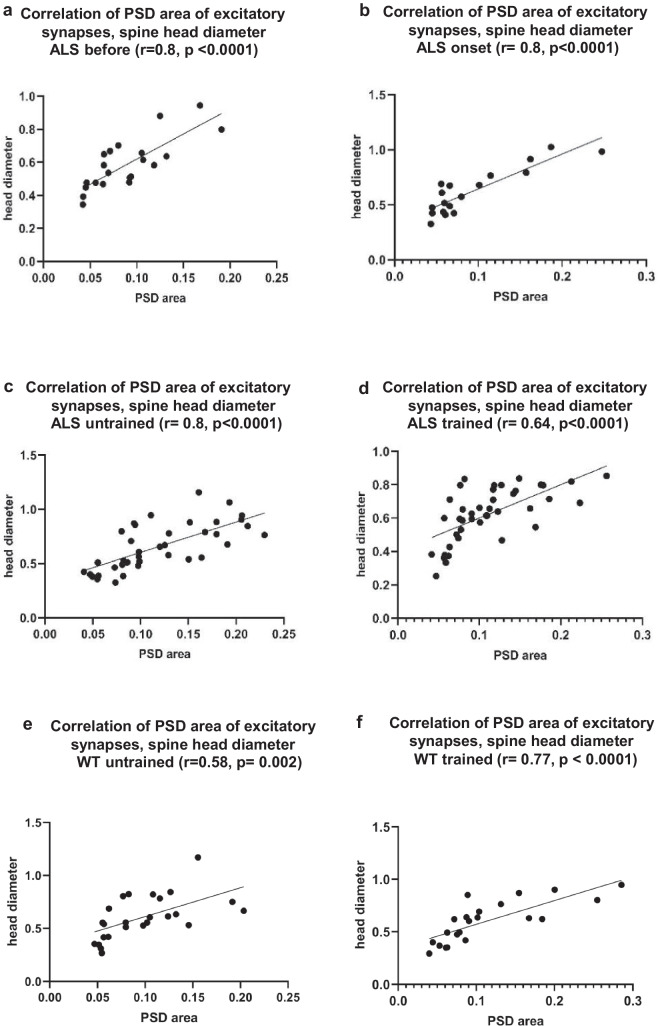
Correlation between PSD area of excitatory synapses and spine head diameter. **a** Pearson correlation between PSD area of excitatory synapses and spine head diameter in the ALS before group (*r* = 0.8, *p* < 0.0001), **b** Pearson correlation between PSD area of excitatory synapses and spine head diameter in the ALS onset group (*r* = 0.8,* p* < 0.0001), **c** Pearson correlation between PSD area of excitatory synapses and spine head diameter in the ALS terminal untrained group (*r* = 0.8, *p* < 0.0001), **d** Pearson correlation between PSD area of excitatory synapses and spine head diameter in the ALS terminal trained group (*r* = 0.64, *p* < 0.0001), **e** Pearson correlation between PSD area of excitatory synapses and spine head diameter in the WT untrained group (*r* = 0.58, *p* = 0.002), **f** Pearson correlation between PSD area of excitatory synapses and spine head diameter in the WT trained group (*r* = 0.77, *p* < 0.0001)

Pearson correlation analysis was performed to assess the relationships between PSD area of both excitatory and inhibitory synapses and the spine head diameter. Positive correlations between the PSD area of excitatory synapse and the diameter of spine heads are as follows; in the ALS before (Pearson correlation coefficient, *r* = 0.8, *p* < 0.0001) (Fig. 4a), ALS onset (Pearson correlation coefficient, *r* = 0.8, *p* < 0.0001) (Fig. 4b), ALS terminal untrained (Pearson correlation coefficient, *r* = 0.8, *p* < 0.0001) (Fig. 4c), and ALS terminal trained (Pearson correlation coefficient, *r* = 0.64, *p* < 0.0001) (Fig. 4d). Significant positive correlations were observed in the WT untrained group (*r* = 0.58, *p* = 0.002) (Fig. 4e) and a stronger association in the WT trained group (*r* = 0.77, *p* < 0.0001) (Fig. 4f).

**Fig. 5:**
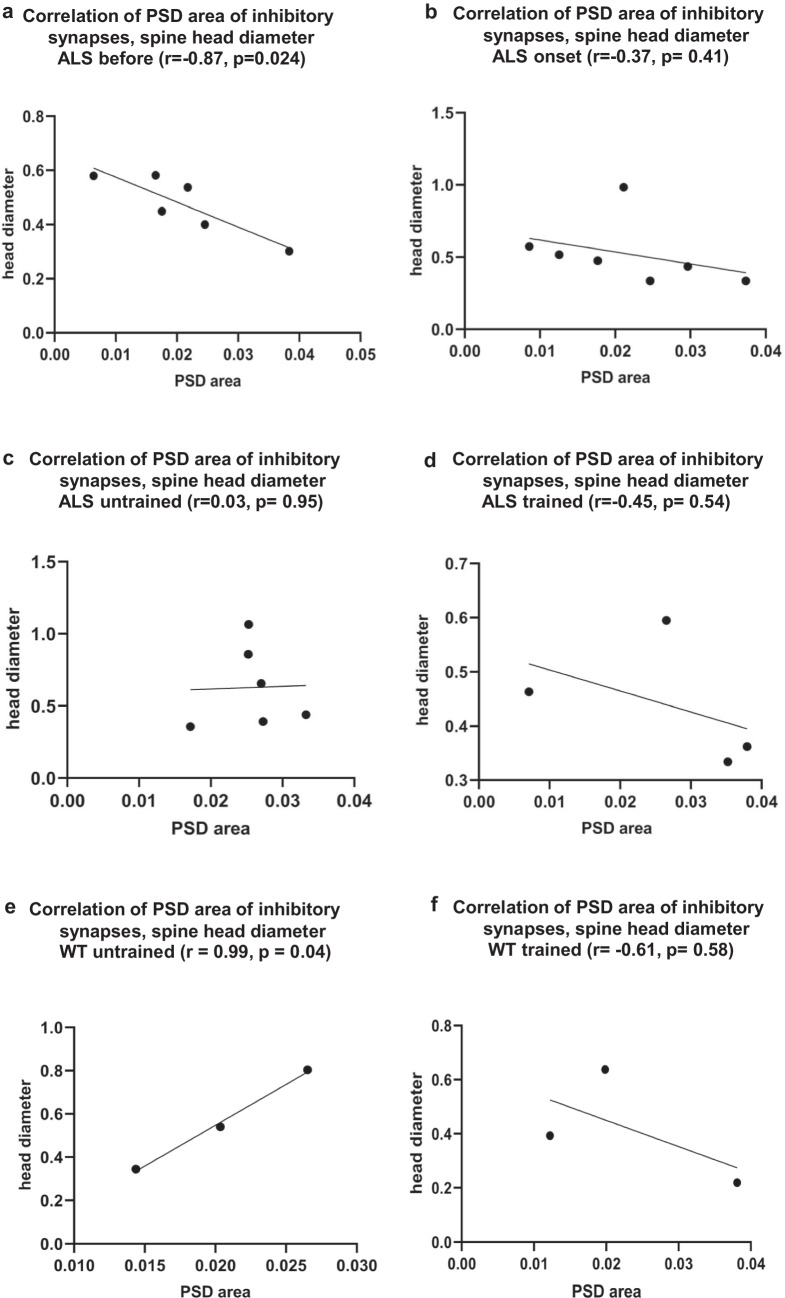
Correlation between PSD area of inhibitory synapses and spine head diameter. **a** Pearson correlation between PSD area of inhibitory synapses and spine head diameter in the ALS before group (*r* = −0.87, *p* = 0.024), **b** Pearson correlation between PSD area of inhibitory synapses and spine head diameter in the ALS onset group (*r* = −0.37, *p* = 0.41), **c** Pearson correlation between PSD area of inhibitory synapses and spine head diameter in the ALS terminal untrained group (*r* = 0.03, *p* = 0.95), **d** Pearson correlation between PSD area of inhibitory synapses and spine head diameter in the ALS terminal trained group (*r* = −0.45, *p* = 0.54), **e** Pearson correlation between PSD area of inhibitory synapses and spine head diameter in the WT untrained group (*r* = 0.99, *p* = 0.04). **f** Pearson correlation between PSD area of inhibitory synapses and spine head diameter in the WT trained group (*r* = −0.612, *p* = 0.58)

The correlation between PSD area of inhibitory synapses and spine head diameters was negative in the groups as follows: ALS before (Pearson correlation coefficient, *r* = −0.87, *p* = 0.024) (Fig. 5a), ALS onset (Pearson correlation coefficient, *r* = −0.37, *p* = 0.41) (Fig. 5b), ALS terminal trained (Pearson correlation coefficient, *r* = −0.45, *p* = 0.54) (Fig. 5d), and WT trained group (Pearson correlation coefficient, *r* = −0.61, *p* = 0.58) (Fig. 5f). In contrast, a weak positive correlation was observed in the ALS terminal untrained group (*r* = 0.03, *p* = 0.95) (Fig. 5c), and a positive correlation was found in the WT untrained group (*r* = 0.99, *p* = 0.04) (Fig. 5e).

#### Shape of Spines

**Fig. 6:**
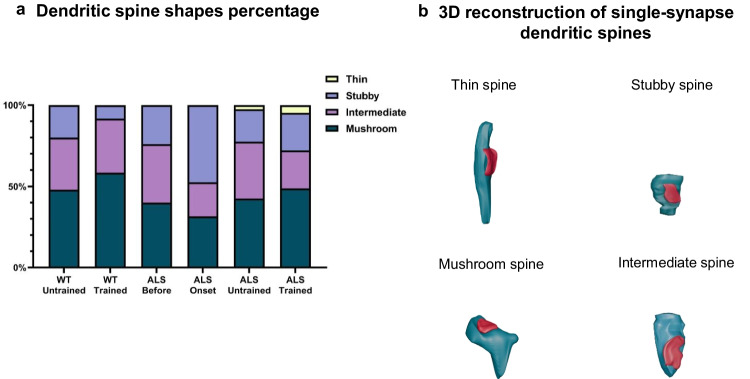
Dendritic spine morphology. **a** Percentage distribution of dendritic spine shapes in WT untrained and trained, ALS before, ALS onset, ALS terminal untrained, and ALS terminal trained groups is shown as a stacked bar chart. Spines were classified as thin, stubby, intermediate, or mushroom. **b** 3D reconstruction of single-synapse dendritic spines showing different spine shapes: thin, stubby, mushroom, and intermediate. Excitatory synapses are marked in red

The distribution and proportions of dendritic spine shapes in the experimental groups were as follows: in the ALS before group were 40% mushroom, 36% intermediate, and 24% stubby. In the ALS onset group, 31.58% mushroom, 21.05% intermediate, and 47.37% stubby spines. In the ALS terminal untrained, 42.5% mushroom, 35% intermediate, 20% stubby, and 2.5% thin spines. In the ALS terminal trained group, 48.83% mushroom, 23.25% intermediate, 23.25% stubby, and 4.65% thin spines. In the WT untrained group, 48% mushroom, 32% intermediate, and 20% stubby spines. In the WT trained group, 58.33% mushroom spines, 33.33% intermediate, and 8.33% stubby spines. Due to the very low number of thin spines, spine shape categories were merged for statistical analysis. Mushroom spines were classified as mature, whereas thin and stubby spines were grouped as immature [5], with intermediate spines analyzed as a separate category. Categorical data analysis revealed no significant differences in the distribution of dendritic spine shapes among the experimental groups (*χ*^2^ = 15.40, df = 15, *p* = 0.42). The 3D reconstructions of the different spine shapes are presented in Fig. 6b and their percentage distribution in each group in Fig. 6a.

## Discussion

To the best of our knowledge, this is the first report describing synaptic plasticity and the quantitative assessment of asymmetric (putative excitatory) and symmetric (putative inhibitory) synapses in the somatosensory (barrel) cortex of female ALS mice during disease progression and after swim training. The somatosensory cortex was selected for this study because of its role in processing sensory feedback and its close functional interaction with motor cortical areas during motor learning and execution.

In this study, we demonstrated the alterations in the B2 barrel of the somatosensory cortex at the pre-symptomatic stage of ALS. These changes included an increase in the density of excitatory synapses in the ALS group before any observable signs of disease, decreases in the excitatory synapse density in the ALS onset and trained groups, and an increase in the density of inhibitory synapses in the early symptomatic stage and in ALS untrained groups, while swim training had no effect on the inhibitory synaptic density.

Our results showed that the number of excitatory synapses was higher in the ALS before group compared to the aged-matched WT group, indicating that the elevated density of excitatory synapses in the barrel cortex starts even before the first signs of the disease. This finding is consistent with somatosensory evoked potentials (SEP) recordings reported by Cengiz et al. [8], which showed increased somatosensory cortical excitability in ALS patients. This could be an important factor in the disease progression since, in the same study, they found that heightened somatosensory excitability was associated with reduced pulmonary function and motor strength [8]. Similarly, the transcranial magnetic stimulation (TMS) recordings made by Menon et al. [36] showed that cortical hyperexcitability is an early feature of ALS, often preceding the onset of muscle weakness and being detectable in asymptomatic carriers of ALS-related gene mutations. The increased number of excitatory synapses observed in the B2 barrel of the somatosensory cortex, potentially reflecting altered excitatory signalling, may be related to the alterations detected by SEP and TMS recordings and contribute to disturbances in motor-related cortical processing and sensorimotor integration. Such alterations in excitatory synapse density and E/I balance, which have been associated with cortical hyperexcitability reported in previous studies using electrophysiological recordings, could interfere with the processing of sensory input required for precise motor control, potentially delaying motor planning and reducing the system’s responsiveness to sensory feedback during movement. However, at disease onset, we observed a decrease in the number of excitatory synapses compared to the ALS before group. This reduction in the number of excitatory synapses could be due to excitotoxicity, involving an excessive release of neurotransmitters such as glutamate, which overstimulates neurons, leading to calcium overload and ultimately neuronal damage and death [54]. Although excitatory synapse numbers decreased during disease progression, measurements showed no changes in the PSD area. This may reflect compensatory mechanisms that help maintain network integrity despite ongoing pathological processes such as protein aggregation, excitotoxicity, or oxidative stress.

Our findings indicate an increase in the density of excitatory synapses in the WT trained group in comparison with the WT onset group, suggesting a possible association between swimming and synaptic organization in WT groups. In the ALS groups, the reduction in excitatory synaptic density in the ALS trained group in comparison with the ALS before group suggests a compensatory reduction in excitatory synaptic connectivity or reorganization of cortical synaptic architecture. Such structural alterations are likely to be relevant to early functional abnormalities, including cortical hyperexcitability, which has been reported as an early feature of ALS [36]. However, further studies are required to determine the functional significance of these ultrastructural changes and to clarify their relationship to cortical network activity.

On the other hand, the inhibitory synapse density was higher in the ALS terminal untrained group than in the corresponding WT untrained group. Both the ALS onset and ALS terminal untrained groups also exhibited increased inhibitory synapse density relative to the ALS before group. These data indicate that the density of inhibitory synapses, which is low before the disease onset, increases with the first symptoms of ALS and remains stable as the disease progresses. While these results are inconsistent with the findings of Höffken et al. [25] and Cengiz et al. [8], where SEP recordings showed a reduction in the inhibition, they may suggest a compensatory mechanism or response to the increased excitatory synaptic density, potentially associated with cortical hyperexcitability observed in electrophysiological recordings, in the somatosensory cortex. These results highlight the need for additional studies to elucidate the molecular mechanisms underlying these changes and to reconcile the apparent discrepancies between electrophysiological recordings and the synaptic quantifications. Our results did not indicate any effect of swim training on inhibitory synapse density.

The findings indicate significant changes in the E/I ratio in the ALS before group compared with the ALS onset group, the WT before group, ALS untrained, and ALS trained groups. These data suggest that the E/I ratio in the ALS before group is higher than in WT before group and the other ALS groups, which is consistent with the higher density of excitatory synapses and a lower density of inhibitory synapses observed in this group. Such imbalances may be associated with cortical hyperexcitability and with impaired homeostatic plasticity that normally stabilizes neural circuits [21]. The disruption of this ratio in the B2 barrel of somatosensory cortex, occurring before the onset of the disease when no symptoms were yet present, may be associated with altered sensorimotor processing and thus could contribute to the characteristic symptoms of ALS, such as muscle weakness and paralysis [30, 38]. Importantly, we observed a significant decrease in the E/I ratio between the WT trained group and the ALS terminal trained group indicating that at the terminal stage, ALS trained animals exhibit a lower E/I ratio than WT trained (Fig. 2d). The E/I ratio in the WT trained group increased together with an increased density of excitatory synapses (Fig. 2b), which may reflect enhanced synaptic plasticity and strengthened glutamatergic inputs which potentially influence sensory processing. In contrast, the opposite trend was observed in the ALS trained group. The reduction in E/I ratio in ALS trained relative to WT trained could result from excitotoxicity, as ALS pathology drives chronic glutamate overload, leading to loss of excitatory synapses which could override exercise benefits. It may also reflect several non-mutually exclusive mechanisms, including compensatory homeostatic synaptic plasticity aimed to maintain network stability in the context of ongoing pathology, preferential degeneration or loss of excitatory synapses, or a relative shift in inhibitory synaptic prevalence. Additionally, swim training may differentially modulate synaptic remodelling in the ALS compared with WT. Importantly, these ultrastructural changes may not directly translate into functional network excitability as synaptic efficacy depends on multiple factors not captured by TEM, highlighting the need for further studies integrating structural and physiological approaches. No detectable differences in excitatory or inhibitory synapse density, or in the synaptic E/I ratio, were observed between ALS terminal untrained and ALS terminal trained groups in the B2 barrel of the somatosensory cortex. These findings may suggest that, at the terminal stage of ALS, swim training no longer markedly alters cortical synaptic organization under the present experimental conditions. However, advanced pathology at the end stage may limit the capacity for further synaptic remodelling. Alternatively, swim training-related effects may occur at earlier stages but are not maintained at the end stage or may be spatially restricted or layer-specific and therefore not captured within the sampled volume of the B2 barrel. It should also be noted that ultrastructural measures of synapse density may not capture changes in synaptic efficacy or other functional properties. Together, these considerations suggest that while no ultrastructural differences in synapse density were observed, functional or microstructural adaptations cannot be excluded and may underlie experience-dependent plasticity in this region.

Quantitative changes in synapses may be accompanied by additional morphological alterations of dendritic spines, which are small, bulbous protrusions extending from the dendritic shaft that constitute the primary sites of excitatory synaptic input. Their shape and size are closely associated with synaptic strength and plasticity, providing important structural indicators of synaptic efficacy and functional state [1, 35]. Spines serve as biochemical compartments, isolating calcium and other signalling molecules to allow for input-specific changes in the synaptic strength. They also play an electrical role, with the spine’s neck potentially filtering or decreasing the voltage that reaches the dendrite and soma [1].

We did not find any differences in the dendritic spines’ head and neck diameters or their lengths between ALS groups. Based on these results, we could hypothesize that this lack of changes in spine morphology is due to either resilience and/or compensatory mechanism aimed at restoring or maintaining synaptic functions, or to the presence of stable but non-functional spines in the somatosensory cortex. Although our results at the ultrastructural levels did not indicate any differences in the spine morphology and measurements in layer IV of the somatosensory cortex, in a study performed by Fogarty et al. [19], who examined spine density in layer V pyramidal neurons of the motor cortex using Golgi-Cox method, they reported progressive decreases in dendritic length and spine density, starting at pre-symptomatic stages. In another study conducted by Costa-Pinto et al. [12], significant changes were found in the pyramidal neurons of the motor cortex, particularly in layer V. These included dendritic regression, a reduction in dendritic spine density, and increased excitatory synaptic currents, all occurring at the pre-symptomatic stage.

The positive correlation observed between the PSD area of excitatory synapses and spine head diameter in ALS groups indicates a direct relationship, meaning that as the PSD area increases, the spine head also becomes larger. This relationship may reflect changes in excitatory synaptic organization or efficiency. In contrast, the PSD area of inhibitory synapses showed a negative correlation with spine head diameter, except in the ALS terminal untrained group, where a very weak positive correlation was observed. These results are consistent with the fact that inhibitory synapses are most often located on the spine neck rather than on the head [32, 33]. In the ALS before group, this negative correlation reached statistical significance, suggesting that as the inhibitory PSD area increased, the spine head diameter decreased. Such a pattern may reflect synaptic dysfunction or changes in spine remodelling that affect the shape, volume, and stability of the spine head. The observed positive correlation between the PSD area of excitatory synapses and spine head diameter in both WT untrained and trained groups indicates a strong structural coupling between excitatory synaptic and dendritic spine morphology in the B2 barrel cortex, with stronger correlation in the WT trained group (Fig. 4e, f). This relationship suggests that spine head size is a reliable morphological correlate of excitatory synaptic strength, and that this structure-structure coupling is maintained, and potentially reinforced, following swimming in the WT group. In contrast, the positive, significant (WT untrained) and negative, non-significant (WT trained) association between PSD area of inhibitory synapses and spine head diameter in WT groups (Fig. 5e, f), indicates that inhibitory synaptic specializations are not consistently linked to spine morphology and are likely regulated independently of spine structural remodelling. Importantly, these correlation patterns align with the absence of changes observed in the synaptic E/I ratio following swimming in WT groups. This suggests that swimming may refine the coupling between excitatory synaptic ultrastructure and postsynaptic spine architecture without shifting the overall excitation-inhibition balance. Together, these findings support a model in which swimming induces subtle, structure-specific adaptations within excitatory synapses, reflected in strengthened PSD spine coupling, while preserving the overall synaptic composition of the circuit. The stability of the E/I ratio further suggests that experience-dependent plasticity in this context is expressed more through fine-scale synaptic organization and structure–function alignment rather than changes in synapse abundance.

These findings should be interpreted with caution, as an increase in excitatory synapse density does not necessarily correspond to increased neuronal excitability. This is particularly important when TEM-based data are considered in isolation, since TEM provides high-resolution visualization of synaptic ultrastructure but does not capture key functional determinants such as synaptic strength, receptor composition (e.g., AMPA/NMDA ratios), or network-level excitation-inhibition balance. Moreover, neuronal excitability is profoundly shaped by intrinsic membrane properties such as ion channel expression (e.g., Na⁺, K⁺, Ca^2^⁺ channels) and dendritic integration, which TEM cannot directly assess despite its precision in structural imaging. Thus, elevated excitatory synapse density often signifies structural plasticity or remodelling without necessarily driving heightened cortical excitability at the functional level.

Since there is currently no known cure for ALS, and as the disease progresses, the therapeutic window for preserving functional motor units becomes increasingly limited, the choice of appropriate physical activity is of great importance. Previous studies have shown that while running and other high-intensity exercise regimens produced inconsistent outcomes, sometimes improving certain parameters but in other cases exacerbating symptoms, swim training has demonstrated more consistent neuroprotective and metabolic benefits. Unlike high-intensity running, which amplifies motor neuron hyperexcitability and glutamate release in SOD1-G93A female mice, moderate swimming has been shown to improve skeletal muscle metabolism, glucose utilization in the SOD1 model, sustain motor function, preserve the BDNF/TrkB pathway, prevent motor neuron loss, and reduce oxidative stress, thereby promoting motoneuron survival [13–15, 18, 29, 44, 45, 50]. Although all these studies and their results seem promising from a therapeutic perspective, very few have investigated the effects of swimming in female mice or compare female and male groups, highlighting an important direction for future research.

While our TEM-based synaptic density quantification did not reveal significant improvements in synaptic ultrastructure (i.e., excitatory synapse density or excitatory-inhibitory ratio) following swim training in the somatosensory cortex of ALS mice, this intervention did not produce any detectable adverse effects. Specifically, ALS trained mice showed no increase in the density of excitatory synapses compared to the ALS untrained group. These ultrastructural outcomes align with swim training’s established safety profile across SOD1-G93A studies. Subsequent studies employing higher training volumes, longitudinal TEM assessments, or complementary techniques like array tomography could clarify whether subtle or stage-specific synaptic remodelling occurs under these conditions.

## Limitations of the Study

One limitation of the present study is that the ultrastructural approach does not allow identification of the presynaptic axonal origin or the postsynaptic neuronal subtype. Therefore, the observed synaptic changes cannot be assigned to specific pathways (e.g., M1–S1 projections) or exclusively to layer IV neurons. A detailed analysis of inhibitory synapses targeting neuronal somata was beyond the scope of the present study. Due to inherent constraints of TEM, including limited sampling area and section orientation, the analysis was primarily focused on neuropil regions. Somatic inhibitory synapses play a key role in regulating neuronal output. Their exclusion may therefore lead to some underestimation of total inhibitory input. Nevertheless, the present approach provides a robust characterization of synaptic organization within the sampled regions. Importantly, the quantitative ultrastructural analysis provides a highly precise and direct assessment of synaptic organization at the nanometer scale, enabling detection of subtle changes in excitatory and inhibitory synapse density. The use of TEM enabled high-resolution, quantitative assessment of synaptic ultrastructure, providing detailed insights that are not achievable with other techniques and representing, to our knowledge, the first such analysis in this context. Another limitation of the present study is that spine-based analyses were conducted using pooled spine data rather than treating the individual animal as the statistical unit. As multiple spines originated from the same animal, this approach may have inflated the effective sample size due to within-animal non-independence. Moreover, the wild-type control mice were not littermates of the SOD1-G93A transgenic mice, which may have introduced differences related to genetic background. Moreover, the exclusive use of female mice limits direct extrapolation of the findings to males and highlights the need for future studies including both sexes. While our findings may suggest an association between swim training and altered synaptic organization in this SOD1-G93A cohort, this interpretation must be considered within the limitations of the study, including sample size, absence of littermate controls, and timing of the training intervention. Additionally, studying neuroplasticity in ALS is challenging due to disease heterogeneity, difficulties in distinguishing compensatory from pathogenic alterations, the limited similarity between animal models and the human condition, and rapid disease progression, which restricts experimental investigations. In mouse models such as SOD1-G93A, the barrel cortex has been largely unexplored, with research focusing primarily on early compensatory projections to the motor cortex rather than on intrinsic plasticity deficits. This represents a notable knowledge gap and study limitation, potentially biasing motor-centric interpretations and overlooking sensorimotor confounds in assays such as swimming tests.

## Conclusion

To the best of our knowledge, this is the first study to demonstrate changes in synaptic density in the ALS animal model during disease progression and after swim training. Thus, we believe that the results of this study may have broader significance, supporting further investigation of appropriately designed physical activity programs as complementary supportive strategies in ALS. These findings also highlight the potential relevance of swim training in the context of ALS and encourage further studies aimed at determining whether such interventions may contribute to maintaining motor function and quality of life in ALS patients.

## Data Availability

The datasets that support the results of this study are available in the Jagiellonian University repository, RODBUK UJ, CC-BY. Additional information is available from the corresponding authors on reasonable request.
